# Craniofacial Morphology in Children with Growth Hormone Deficiency and Turner Syndrome

**DOI:** 10.3390/diagnostics10020088

**Published:** 2020-02-07

**Authors:** Dorota Wójcik, Iwona Beń-Skowronek

**Affiliations:** 1 Department of Dental Prosthetics, Medical University of Lublin, 20-081 Lublin, Poland; 2 Department of Paediatric Endocrinology and Diabetology with Endocrine—Metabolic Laboratory, Medical University of Lublin, 20-093 Lublin, Poland; skowroneki@interia.pl

**Keywords:** growth hormone deficiency, Turner syndrome, craniofacial abnormalities, growth hormone, orthodontics, dentistry craniofacial morphology, children, short stature

## Abstract

The review aims to collect and demonstrate recent knowledge about craniofacial morphology in growth hormone (GH)-deficient children and children with Turner syndrome. The review describes also the effects of growth hormone treatment on craniofacial morphology of children with growth hormone deficiency and Turner syndrome. Regardless of the disorder it accompanies, short stature is associated with similar craniofacial features characteristic of all short-statured children. Characteristic craniofacial features involve lesser dimensions of the cranial base and mandibular length, proportionately smaller posterior than anterior facial height, retrognathic face, and posterior rotation of the mandible. We also analyze orthodontic treatment in children affected by disorders associated with GH deficiency or provided with growth hormone treatment in the aspect of craniofacial growth. Recent publications show also the connection between growth hormone receptor polymorphism and craniofacial growth. Specialists and orthodontists treating short-statured children must be aware of the results of studies on craniofacial morphology and educate themselves on the topic of craniofacial growth in children with short stature. Moreover, knowledge of the influence of GH therapy on growth of craniofacial structures is necessary to decide the proper timing and planning of orthodontic treatment.

## 1. Introduction

Child-like craniofacial proportions have been observed in patients with growth hormone deficiency (GHD) for ages. The first reports on the dentition and morphology of children with GHD began to appear in the dental literature in the 1930s and 1940s. These reports, based on individual cases, described the delayed teeth and facial development of children with GHD. Later, larger groups of children with GHD were analyzed for changes in dentition and craniofacial morphology—delayed development of teeth, face, and cranial base were described [1]. Although little is known about the mechanism of action of growth hormone (GH) on craniofacial components, it is believed that therapy with recombinant human growth hormone (rHGH) acts mainly on those craniofacial parts where epiphyseal ossification takes place and on the areas that adapt to this growth—mandibular rami in particular [2]. GH is a peptide hormone excreted by somatotropic cells of the anterior pituitary under control of hypothalamic hormones and ghrelin, as well as other substances produced in the central nervous system [3]. It exerts many actions in the body. It regulates many metabolic pathways. Some of its effects are mediated through insulin-like growth factor-I (IGF-I). GH and IGF-I play important roles in the stimulation of growth and bone metabolism especially with regard to increase of bone mass. In childhood, accumulation of bone mass is a combined effect of bone growth and bone remodeling. In cranial bones, bone growth primarily occurs at the epiphyseal growth plates. It results from differentiation and proliferation of chondrocytes. GH influences these chondrocytes directly, but it mainly regulates this function through IGF-I, which stimulates proliferation and production of the matrix by these cells. IGF1 is produced under GH control in the liver and directly in the cartilage. Bone remodeling is a process in which new bone is formed by osteoblasts while osteoclasts resorb bone. Osteoblastic cells (osteoprogenitor cells) originate from skeletal stem cells with osteogenic differentiation potential, that is, skeletal mesenchymal stem cells (MSCs). MSCs reach bone surfaces from circulation through vascular channels associated with bone remodeling sites [4]. With their arrival at the bone surface, osteoblastic cells produce bone matrix, which becomes mineralized. As far as the old osteoblasts are concerned, they die by apoptosis or become embedded in bone matrix as osteocytes. Chondrocytes and osteoblasts have receptors for GH; administration of GH at physiological doses influences directly osteoblasts, as it stimulates cell proliferation and differentiation [5]. With this mechanism, GH stimulates osteoblast proliferation and activity, and it promotes bone formation directly and through IGF-I. IGF-1 promotes osteoblastogenesis and reduces osteoblast apoptosis [6].

**Figure 1:**
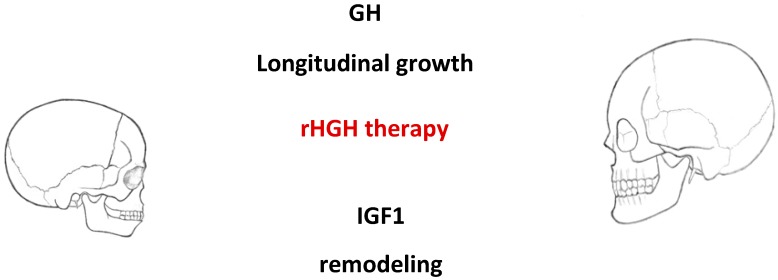
Association between longitudinal cranial bone growth, remodeling, and rHGH therapy. The therapy of rHGH impacts the growth of the skull in a dual mechanism: by longitudinal growth of bones under GH influence and by remodeling of bones under IGF1 action.

These hormones also stimulate osteoclast activity and differentiation, thus promoting bone resorption. Osteoclasts express IGF-1 receptors and IGF-1 influences directly their function [7]. During bone formation, numerous proteins, including TGFβ, BMPs, FGFs, and IGFs, are released subsequently by osteoclasts during resorption when they can become activated and available locally to promote the resorptive process. In vitro, IGF-1 induces RANK-L (receptor activator of nuclear factor κB) synthesis in precursors of osteoclasts and, as a consequence, is involved in osteoclastogenesis [8]. IGF-I is required for maintaining the normal interaction between the osteoblast and osteoclast to support osteoclastogenesis through its regulation of RANKL and RANK expression. The stimulatory effects of IGF-1 on bone resorption may be explained by the induction of RANK-L by IGF-1. The induction of osteoprotegerin by GH may, on the other hand, temper these effects [9]. This results in an increase in the overall rate of bone remodeling, with a net effect of bone accumulation [10,11,12]. In case of no GH, the rate of bone remodeling is reduced and there is a gradual loss of bone mineral density [13]. The deficiency of GH severely limits bone growth and the accumulation of bone mass. According to the recent preliminary studies, there is a possible application of GH in bone regeneration [14] (Figure 1).

## 2. Literature Review

### 2.1. Craniofacial Morphology in Children with GHD and IGHD

Mechanisms that control development of facial bones involve complex interactions between genes, hormones, and nutrition that affect the final shape of facial bones. If these mechanisms become disrupted, it may lead to an unbalanced growth model. Short stature is often a consequence of an unbalanced growth model, which may also affect growth of facial bones. Comparisons between children with IGHD (isolated growth hormone deficiency) and those with GHD demonstrate rather similar characteristics in both groups [15,16].

Delayed growth does not affect facial structures to the same extent, resulting in abnormal face morphology. Most studies report mandibular retroposition, wide gonion angle, and relatively increased lower facial height. The maxilla seems to be affected to a lesser extent than the mandible, although some studies show its retroposition [1]. Disproportionate growth of the structures of the cranial base and jaws results in retrognathia, proportionally smaller lower facial height and sharp vertical inclination of the mandible. Researchers wonder if delayed growth affects all of the facial structures, as the maxilla is generally affected to lesser extent than the mandible. Children with GHD are described in the literature as possessing a large skull and a childish face that contrasts with their intelligence, which remains within normal limits for their age. Children with GHD are shorter than their peers and possess a thicker layer of subcutaneous adipose tissue. Cephalometric studies demonstrated reduced dimensions of the anterior and posterior cranial bases and smaller mandible [17]. Similarly, Pirinen’s analysis of children with hypopituitarism demonstrated lower cranial base dimension, small posterior facial height, and small posterior mandibular height, even when GHD children were compared to healthy children of the same height. Among patients with hypopituitarism, not treated with GH, upper face height (N-ANS) was the most reduced dimension compared to children of the same age [1]. Similar features of craniofacial morphology may be found in children with GHD. Girls with GHD have significantly smaller mandibles, retrognathic maxillae, and mandibles, while boys are characterized by flat cranial base, both jaws retropositioned, retroinclined mandible, and underdeveloped cranial base, maxilla, and mandible [18,19]. Moreover, they often require orthodontic treatment due to higher incidence of crowding among children with short stature [15]. The newest review by Davidopoulou et al. demonstrated that regardless of the cause of growth retardation in children with IGHD and GHD, they possess similar characteristics of craniofacial morphology, such as short length of the cranial base and the mandible, increased lower facial height, retropositioned mandible, and wide gonion angle [20].

Growth of facial bones is unpredictable following GH administration. However, facial convexity decreases, mandibular length increases, and posterior facial height increases. Choi et al. found that before GH treatment, boys with GHD had shorter anterior and posterior cranial base length, mandibular ramus height, and corpus length compared to the control group. On the other hand, girls with GHD had shorter anterior cranial base length and mandibular ramus height [16].

### 2.2. Craniofacial Morphology in Children with IGHD

There can be isolated growth hormone deficiency (IGHD) or growth hormone deficiency in combination with other general disorders. IGHD mutations in the genes encoding GH (GH1) can lead to classic GHD (types IA, IB, and II) or a biologically inactive GH syndrome [21]. The clinical features of patients with IGHD may be different, depending on the age at onset, severity of the disorder, or etiology. Patients who suffer from congenital IGHD show characteristic growth patterns; they display significant maturational delays and reduced somatic growth. Infants with congenital IGHD usually present with normal birth length and weight. Their growth is normal for 3-6 months. However, linear growth rates decelerate afterwards. Linear growth curves in those cases deviate from the mean progressively [22].

In children with IGHD, length and convexity of the face are disproportionately small in relation to age, their faces retaining the childish convexity. Numerous studies demonstrated that the total height of the face (Gn-Cd) is reduced, primarily as a result of reduced height of the mandibular ramus (Cd-Go). [18] Moreover, the maxilla is significantly smaller and relative reduction of mandibular size may also be present [23,24]. The maxilla is often retrognathic, although usually to a lesser extent than the mandible [1,25]. Many studies report that the length of the posterior cranial base is smaller than that of the anterior (N-S) cranial base [23,25]. On the other hand, facial convexity becomes reduced with GH replacement therapy and growth of the condylar processes of the mandible seems to be its main effect [26]. Another study demonstrates that growth in the Gn-Cd dimension and increase in the lower facial height (Ans-Me) are accelerated, while the length of the cranial base changes minimally [23]. Cantu et al. showed that correcting growth deficiency during GH therapy affects anterior facial height, posterior facial height, and posterior cranial base [25]. Funatsu et al. determined the effects of GH therapy on craniofacial growth in children with IGHD. Fifty-seven patients treated for GHD were included and subsequently divided into three groups depending on the length of GH treatment: untreated patients, patients treated for a short period of time (mean time of GH replacement therapy 1.2 years), and patients undergoing long-term treatment with GH (mean time of GH replacement therapy over 2 years). Since the greatest growth during GH treatment is observed during the first two years of therapy, researchers divided patients into short-term vs. long-term treatment depending on the two-year treatment period. In the untreated group, anterior cranial base and height of the ramus were lower than standard values. They compared mean values for patients with IGHD and standard values for individuals of the same sex and similar chronological age in the particular age group. In boys, ANS-Me, mandibular length (A-Ptm), Gn-Cd, mandibular body length (Pog-Go), and Cd-Go were significantly smaller compared to standard values. In the group of boys on short-term treatment, the Cd-Gn dimensions were significantly greater, while SNA and mandibular angle were significantly smaller compared to standard values.

Compared to the untreated group, patients on long-term GH treatment were characterized by significantly greater upper facial height, length of the maxilla, and height of the mandibular ramus. Children on long-term GH replacement therapy presented with increased growth of the facial part of the cranium, especially the maxilla and mandibular rami. Among boys treated with GH for longer than two years, the total facial height dimensions (N-Me), ANS-Me, A-Ptm, Pog-Go, and Cd-Go were significantly smaller compared to standard values. The plane of the mandibular base was flat and the mandibular angle was significantly smaller. Among girls, ANS-Me, Cd-Go, and gonion angle were significantly smaller, while A-Ptm was significantly greater than standard values [18].

The literature contains several reports describing growth characteristics of the facial part of the cranium in cases of untreated IGHD. Cantu et al. compared groups treated with GH and untreated. However, other authors studied patients with IGHD without the distinction of groups depending on GH treatment [25]. With regard to the N-S dimension, Poole et al. described lack of shortening, while Cantu et al. reported reduction by 0.15 SD compared to standard values [23]. There are many sites of intracartilaginous growth at the base of the skull. Although it is given that GH accelerates intracartilaginous growth, there is little evidence to support these statements [5,27]. Research shows that A-Ptm is significantly reduced to the same extent as the mandible [23,24]. Although the maxilla is often positioned posteriorly, A-Ptm dimension is reduced no more than the mandible [1,25]. As far as the mandible was concerned, the SD scores in the untreated group were as follows: −0.71 (Gn-Cd), −0.86 (Cd-Go), and −0.72 (ANS-Me) (i.e., all lower than the respective standard values). This is in agreement with the literature [1,23,24,25].

### 2.3. Effects of GH in Children with IGHD

GH therapy accelerates cartilaginous growth [5,27]. As a result, the growth rate of bones developed through intramembranous ossification, including cranial sutures, as well as endochondral ossification, may increase. Accelerated endochondral growth was evidenced by increased Cd-Go dimension, which is a vertical component. It may be related to the effect of GH, which plays a significant role in bone growth in length. 

### 2.4. Mean SD for N-ANS, A-Ptm, and Cd-Go Following Administration of GH

There were no statistically significant differences between studied groups beside Gn-Cd and Pog-Go. However, mean SDs exhibited growing tendency with longer time of GH therapy [18]. Rongen-Westerlaken et al. determined that mandibular angle grew due to a significant increase in Cd-Go and thus, ANS-Me increased significantly at the same time. On the other hand, Funatsu et al. did not observe any significant increase in ANS-Me growth during GH therapy [18,28].

These findings suggest that GH accelerates craniofacial development. Moreover, the effect of GH therapy on final height depends on the time of GH therapy, dose, frequency of GH administration, and age of commencing GH treatment. There are no studies showing how time of GH therapy influences craniofacial morphology. However, Funatsu et al. attempted to investigate the influence of GH treatment on craniofacial morphology in children with IGHD depending on short-term vs. long-term GH administration. Children receiving long-term growth hormone replacement therapy (over two years) experience increased craniofacial growth, particularly with regard to the height of the mandibular ramus [18,29].

### 2.5. Turner Syndrome 

Turner syndrome (TS) is a relatively common disorder. It occurs in 1:25,000 female births and follows from complete or partial absence of the X chromosome. There are also rare cases in which X chromosomes are structurally abnormal. Apart from the main characteristic (i.e., short stature), cranial growth reduction has been reported too. Although GH deficiency is not usually present in TS, the GH–insulin-like growth factor (IGF)–IGF binding protein (IGFBP) axis is disturbed. Increased IGFBP-3 proteolytic activity has been found in adult TS combined with low levels of circulating free IGF-I and increased levels of free IGF-II. Normal formation of the 150 kDa IGFBP-3 ternary complex (acid-labile subunit (ALS), IGF-I, and IGFBP-3) is also disrupted in TS, together with decreased levels of intact IGFBP-1, -2, and -4, and decreased bioactive IGF-I. This is all in spite of normal immunoreactive levels of total IGF-I, IGFBP-1, -2, -3, and -4, and ALS [30]. A sex-hormone-dependent IGFBP-3 protease (serine protease) leads most probably to destabilization of the 150 kDa IGFBP-3 ternary complex in TS since hormone replacement therapy (HRT) with estrogen leads to substantial reduction in IGFBP-3 proteolysis, which suggests modulation of the protease by sex hormones. At the same time, after HRT in TS, free IGF-II decreases while free IGF-I remains unchanged. In response to HRT, the binary complex of IGF-I and IGFBP-1 increases and the proteolysis of IGFBP-3 normalizes, leading to normalization of ternary complex formation [31]. According to a study from 1997, haploinsufficiency for the short stature homeobox-containing gene (SHOX) also contributed to the marked short stature in TS [32,33]. The SHOX gene is located on the distal end of the X and Y chromosomes at Xp22.3 and Yp11.3, respectively, in the pseudoautosomal region [34]. It regulates chondrocyte proliferation and differentiation and it encodes a cell-type specific nuclear transcriptional activator, which is expressed mostly in osteogenic cells. SHOX acts as a repressor of fusion of the growth plate as it is specifically expressed in the growth plate in hypertrophic chondrocytes undergoing apoptosis. The haploinsufficiency of the SHOX gene can result in premature growth plate fusion in the distal limbs in TS. It also influences the short stature of XO females as two copies of this gene are required for normal height [34,35]. A wide spectrum of abnormalities is associated with mutations or deletions of the SHOX gene. They range from short stature without dysmorphic abnormalities to a form of short stature characterized by disproportionate shortening of lower legs and forearms, Madelung deformity, cubitus valgus, short fourth metacarpals, and high-arched palate [36].

Rongen et al. examined children with Turner syndrome treated with GH and demonstrated statistically significant growth of mandibular rami compared to a cross-sectional control group. Growth was associated with the height of the mandibular rami, not the length of the mandible, maxilla, or anterior cranial base [28]. Juloski et al. demonstrated that patients treated with pharmacological doses of GH were characterized by significantly greater linear dimensions of craniofacial structures than untreated patients. Pharmacological doses are higher than doses used during substitution therapy. Pharmacotherapy (not substitution) with GH mainly affected the posterior face height, mandibular ramus height, total mandibular length, anterior face height, and maxillary length [17]. Craniofacial morphology in patients with Turner syndrome was the subject of extensive investigation. The following cephalometric characteristics were identified: shorter and flattened cranial base and underdeveloped, retrognathic, posteriorly inclined maxilla and mandible [28,37,38,39,40,41]. However, the literature contains reports of normal jaw development [42,43]. GH pharmacotherapy administered for at least two years has a positive effect on the size of the cranial base (mostly anterior cranial base). Earlier studies failed to show this association. There is no effect on shortened posterior cranial base, although that is related to the lack of the X [17].

Studies by Juloski et al. demonstrated that GH also exerts positive effect on maxillary growth. Apparent increase in maxillary length does not, however, result in proper positioning (protrusion), in accordance with other studies [44,45]. Even though an increase in linear dimensions was evident, no statistically significant changes in angular measurements or facial height ratio were observed. No features characteristic for acromegaly were noted. Long-term GH pharmacotherapy in patients with Turner syndrome has a positive effect on the development of the craniofacial complex. The greatest effect of GH administration is observed with regard to posterior facial height and mandibular ramus. However, GH is not able to compensate for the absence of the X chromosome and balance the craniofacial features. It should be also emphasized that sagittal and vertical position of the maxilla was slightly improved, which might suggest a positive effect of GH on its position. Further studies are necessary to determine whether proper position and dimensions of the jaw bones are achievable in children with TS. Despite positive effect on craniofacial growth, it is not possible to eliminate craniofacial features typical for TS using GH pharmacotherapy [17].

### 2.6. Orthodontic Treatment in Children Affected by Disorders Associated with GH Deficiency in the Aspect of Craniofacial Growth—Review of Case Reports

Effects of growth hormone on craniofacial bones are poorly elucidated. It is believed that GH therapy affects facial bones, especially in regions where cartilage-mediated growth takes place, such as the mandibular head or ramus. In patients with GH deficiency, replacement therapy can accelerate development of craniofacial structures. The literature reports cases of patients with Turner syndrome where GH replacement therapy combined with orthodontic treatment led to significant elongation of the mandible. There are also reports of treatment of short-statured teenagers without GH deficiency. Since these patients had class III, accelerated mandibular growth caused by GH treatment hindered orthodontic treatment and worsened patients’ profiles. Previous studies demonstrated that GH therapy might significantly augment mandibular growth, especially of the ramus and condyle. It seems that GH contributes to the change in mandibular size [15]. Previous long-term studies by Kjellberg and Wikland demonstrated that GH treatment brings about a more pragmatic growth model in cases of GH deficiency and idiopathic short stature without GH deficiency [19]. The Division of Pediatric Endocrinology and Metabolism, Seoul National University College of Medicine noted that GH therapy in class II patients with idiopathic short stature tends to worsen facial asymmetry in some patients. Facial asymmetry in those patients should be carefully assessed during GH treatment. Further studies are necessary to establish a proper dose for improvement of mandibular growth in class II patients and to assess for possible side effects of therapy [2]. Hwang and Cha described a case of a short-statured girl treated with GH during orthodontic treatment. The patient described by Hwang and Cha had class III with anterior crossbite, a feature characteristic for Asians. In the study assessing the relative position of molar teeth in short-statured children, 22%–25% of patients had class II defects with large horizontal overjet, although class I defects were most common. However, in the described case, facial profile worsened despite correction of anterior crossbite [15].

Previous studies demonstrated that GH affects the condyle of the mandible and induces transverse growth of the mandibular ramus, leading to a change in direction of mandibular growth from posterior to anterior. Since it is difficult to predict the direction of mandibular growth, it may cause problems in children with large mandibular plane angles and relatively normal size. Orthodontists should take into consideration and understand the characteristics of craniofacial bones in short-statured children before implementing orthodontic treatment and how differences between calendar age and bone age might impact the timing and methods of orthodontic treatment. If a short-statured child is undergoing GH therapy, commencement of orthodontic treatment after finishing therapy with GH should be considered. Unconsidered treatments should be avoided at that time due to unpredictable growth of the mandible. Orthodontists should be advised to delay orthodontic treatment of short-statured children with anterior crossbite until GH therapy is finished. However, if the treatment has been commenced after all, it should be taken under consideration that GH replacement therapy affects mandibular growth more than it affects development of maxilla. Also, both the pattern as well as the degree of growth during GH therapy is difficult to predict and GH rarely affects maturation of teeth [2,46,47].

Structured prospective cohort studies are necessary to assess whether GH treatment could significantly induce development of the mandible in class II patients and act synergistically with orthodontic treatment. However, even if GH treatment could induce mandibular growth in class II patients, the high cost of therapy and high probability of side effects, such as type II diabetes, could raise concerns with regard to applying GH treatment in class II patients without GH deficiency. Results suggest that GH accelerates craniofacial growth, thus improving teeth occlusion and facial profile in some patients. In orthodontics, growth factors such as GH could be useful for correction of class II mandibular retrusion [18]. Particular care should be taken in class III malocclusion or facial asymmetry in patients treated orthodontically during GH therapy. It is believed that body height during adolescence correlates with final body height. In order for GH therapy to be successful, growth should be stimulated before adolescence in order to achieve height close to normal before children reach adolescent age. This principle could also be applied to craniofacial growth, especially in the intramembranous growth zones. Further studies on that topic as well as studies on calvarial length are necessary [18].

## 3. Future Perspectives—Growth Hormone Receptor Polymorphism and Craniofacial Morphology 

Craniofacial morphology is a multigene, quantitative characteristic determined by genetic and environmental factors. Studies assessing craniofacial similarities between close relatives showed that genetic factors play an important role in determining craniofacial morphology. Moreover, comparisons involving monozygotic and dizygotic twins demonstrated clear impact of genes on craniofacial morphology. Identifying the susceptibility of genes on a specific craniofacial phenotype could allow for more effective diagnosis and treatment of congenital craniofacial anomalies, such as mandibular prognathism [48]. The latest reports indicate that growth hormone receptor single nucleotide polymorphisms are associated with mandibular height in Japanese and Chinese populations [49,50]. P561T polymorphism in exon 10 of growth hormone receptor coding gene is related to mandibular height in the Japanese population. There are two isoforms of GHR in humans, generated by retention or exclusion of exon 3 during splicing: a full-length isoform and an isoform that lacks exon 3 (d3-GHR). GHR polymorphism results in deletion of exon 3, which is associated with strong response to growth hormone (i.e., children who are carriers of at least one d3-GHR allele are characterized by 1.7–2-times greater response to growth hormone compared to fl-GHR/fl-GHR homozygotes) [51]. This common polymorphism of human GHR, which results in exon 3 loss, was recently tied to augmented response to GH treatment in French children with short statures who were born small for their gestational age or with idiopathic short stature (IGHD), as well as German children with Turner syndrome or Brazilian children with growth hormone deficiency [36,51,52]. Understanding the ethnic differences with regard to the relationship between GHR and mandibular ramus height may be the key to determining the prevalence of alleles in a multiethnic population. Kang et al. investigated growth hormone receptor polymorphism, in particular, the relationship between GHR polymorphism (d3/fl-GHR) that results in deletion of exon 3 and GHR genotype in exon 10, and craniofacial morphology in the Korean population. They demonstrated that GHR polymorphism (d3-fl-GHR) had no association with examined craniofacial dimensions (cranial base length, maxillary length, overall mandibular length, mandibular corpus length, and mandibular ramus height), but there was a statistically significant relationship between GHR P561T and C422F polymorphism in exon 10 and mandibular height. Taking all this into account, we believe that there is a significant association between the C422F and P561T polymorphisms of GHR (present in LD) and mandibular ramus height in the Korean population. There are very few studies examining the relationship between craniofacial morphology and GHR gene polymorphism [48].

Dos Santos et al. discovered a relationship between d3-GHR genotype and augmented response to large doses of rhGH in children with short stature without GH deficiency [51]. Kang et al. found the d3-GHR allele in 15–50% of examined subjects from the Korean population. In the European population, the prevalence of this allele ranges from 25% to 32% and homozygosity is found in 9%–14%. Kang et al. found no association between d3/fl-GHR polymorphism and linear craniofacial dimensions, although it is perplexing whether such a relationship is also present in the European population due to the presence of different ethnical morphologies of the facial part of the cranium [48].

**Figure 2:**
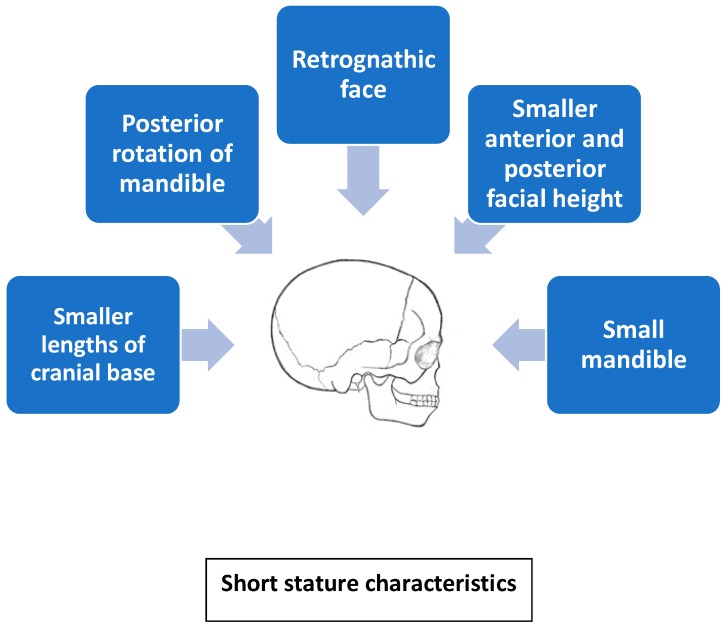
Short stature characteristics.

GH replacement therapy is common in children suffering from its deficiency and the effects of GH on craniofacial growth have been extensively studied. It was demonstrated that GH has an impact on the overall development of craniofacial bones, although it affects some parts of the face more than others. GH exhibits a positive effect on growth of the cranial base, maxilla, and mandible, especially its ramus, as well as on an increase of facial height, which may result in long facial profile and transverse mandibular hypertrophy. Based on previous studies, it can be concluded that GH replacement therapy may partially correct facial profile and teeth occlusion. However, there are also concerns that long-term treatment with GH could lead to development of features characteristic of acromegaly, such as increased dimensions of the feet, hands, or mandible. It may also have a negative effect on facial profile and orthodontic treatment in the case of facial asymmetry or mandibular prognathism. All studies consistently demonstrate that mandibular length depends mainly on growth of its rami. In turn, growth of mandibular ramus leads to anterior rotation of the mandible. Some authors suggest the necessity of paying particular attention during long-term GH therapy, as there are concerns that it could lead to mandibular overgrowth and prognathic malocclusion [28,44] (Figure 2).

## 4. Conclusions 

In conclusion, regardless of the disorder it accompanies, short stature is associated with similar craniofacial features characteristic of all short-statured children. Characteristic craniofacial features involve lesser dimensions of the cranial base and mandibular length, proportionately smaller posterior than anterior facial height, retrognathic face, and posterior rotation of the mandible. Reports concerning mandibular length are contradictory. Moreover, such abnormalities as slightly retarded dental maturity and eruption, tooth crowding, anterior open-bite tendency, and high incidence of distal bite have been reported in children with short stature. The exact amount and pattern of growth after GH administration is unpredictable; however, facial convexity decreases, mandibular length increases, and posterior facial height increases, while tooth eruption remains unaffected. 

Even though the growth model might be a primary factor influencing treatment failure, accelerated mandibular growth and greater increase in height caused by GH therapy could also influence orthodontic treatment. In order to properly plan orthodontic treatment, one must take into consideration the effects of GH therapy on the craniofacial complex as well as assessment of dental age, calendar age, and delayed bone age. 

It is very important that specialists and orthodontists treating short-statured children should be aware of the results of studies on craniofacial morphology and educate themselves on the topic of craniofacial growth in children with short stature. Moreover, knowledge on the influence of GH therapy on growth of craniofacial structures is necessary to make a decision regarding proper timing and planning of orthodontic treatment.

- Children subjected to long-term GH therapy (i.e., more than two years) showed augmented growth of the craniofacial skeleton, especially the maxilla and mandibular rami.
- According to these findings, GH accelerates craniofacial development, improving occlusion and facial profile.
- Treatment with human recombinant growth hormone positively influences longitudinal growth and bone remodeling. The period of treatment seems to be a good time for orthodontic treatment in children with some orthodontic anomalies.
- Long-term GH therapy in patients with Turner syndrome has a positive effect on craniofacial development; GH exerts the strongest effect on posterior facial height and mandibular ramus. However, GH is not able to compensate for the absence of the X chromosome and correct craniofacial features.

## Abbreviations

GHD	growth hormone deficiency
rHGH	recombinant human growth hormone
GH	growth hormone
IGF-I	insulin-like growth factor-I
MSCs	mesenchymal stem cells
TGFβ	*transforming growth factor* *β*
BMPs	*bone morphogenetic protein*
FGFs	*fibroblast growth factor*
RANK-L	receptor activator of nuclear factor κB
IGHD	Isolated GHD
N-ANS	upper face height—linear measurement from nasion to anterior nasal spine
Gn-Cd	total height of the face—gnathion–condylion
Cd-Go	height of the mandibular ramus—condylion–gonion
N-S	anterior cranial base—nasion–sella
ANS-Me	lower facial height—anterior nasal spine–menton
A-Ptm	mandibular length—subspinale–pterygomaxillary fissure
Pog-Go	mandibular body length—pogonion–gonion
SNA	angle between sella, nasion, and subspinale point A
N-Me	total facial height dimensions—nasion–menton
N-ANS	upper anterior facial height—nasion–anterior nasal spine
TS	Turner syndrome
IGF	insulin-like growth factor
IGFBP	IGF binding protein
ALS	acid-labile subunit
HRT	hormone replacement therapy
SHOX	short stature homeobox-containing gene
P561T	P561T heterozygous missense mutation in the growth hormone receptor
d3-GHR	d3-growth hormone receptor, an isoform of GHR that lacks exon 3
LD	linkage disequilibrium

## References

[1] Pirinen S. (1995). Endocrine regulation of craniofacial growth. Acta Odontol. Scand..

[2] Jung M.-H. (2017). Fixed-functional appliance treatment combined with growth hormone therapy. Am. J. Orthod. Dentofac. Orthop..

[3] Devesa J., Almengló C., Devesa P. (2016). Multiple Effects of Growth Hormone in the Body: Is it Really the—Hormone for Growth?. Clin. Med. Insights: Endocrinol. Diabetes.

[4] Andersen T.L., Sondergaard T.E., Skorzynska K.E., Dagnaes-Hansen F., Plesner T.L., Hauge E.M., Plesner T., Delaisse J.-M. (2009). A Physical Mechanism for Coupling Bone Resorption and Formation in Adult Human Bone. Am. J. Pathol..

[5] Ohlsson C., Bengtsson B., Isaksson O.G.P., Andreassen T.T., Slootweg M.C. (1998). Growth hormone and bone. Endocr. Rev..

[6] Krishnan V. (2006). Regulation of bone mass by Wnt signaling. J. Clin. Investig..

[7] Hou P., Sato T., Hofstetter W., Foged N.T. (1997). Identification and Characterization of the Insulin-like Growth Factor I Receptor in Mature Rabbit Osteoclasts. J. Bone Min. Res.

[8] Niu T., Rosen C.J. (2005). The insulin-like growth factor-I gene and osteoporosis: A critical appraisal. Gene.

[9] Mazziotti G., Bianchi A., Bonadonna S., Cimino V., Patelli I., Fusco A., Pontecorvi A., De Marinis L., Giustina A. (2008). Prevalence of Vertebral Fractures in Men with Acromegaly. J. Clin. Endocrinol. Metab..

[10] Mrak E., Villa I., Lanzi R.T., Losa M., Guidobono F., Rubinacci A. (2007). Growth hormone stimulates osteoprotegerin expression and secretion in human osteoblast-like cells. J. Endocrinol..

[11] Menagh P.J., Turner R., Jump D.B., Wong C.P., Lowry M.B., Yakar S., Rosen C.J., Iwaniec U.T. (2009). Growth Hormone Regulates the Balance Between Bone Formation and Bone Marrow Adiposity. J. Bone Miner. Res..

[12] Olney R.C. (2003). Regulation of bone mass by growth hormone. Med. Pediatr. Oncol..

[13] Feingold K., Anawalt B., Boyce A., Chrousos G., Dungan K., Grossman A., Hershman J., Kaltsas G., Koch C., Kopp P. (2000). Disorders of Growth Hormone in Childhood—Endotext.

[14] Sinona B., Franceca G., Alessandro R., Isabella V. (2019). The Role of Growth Hormone in Mesenchymal Stem Cell Commitment. Int. J. Mol. Sci..

[15] Hwang C.-J., Cha J.-Y. (2004). Orthodontic treatment with growth hormone therapy in a girl of short stature. Am. J. Orthod. Dentofac. Orthop..

[16] Choi S.-H., Fan D., Hwang M.-S., Lee H.-K., Hwang C.-J. (2017). Effect of growth hormone treatment on craniofacial growth in children: Idiopathic short stature versus growth hormone deficiency. J. Formos. Med. Assoc..

[17] Juloski J., Dumančić J., Šćepan I., Lauc T., Milašin J., Kaić Z., Dumić M., Babić M. (2016). Growth hormone positive effects on craniofacial complex in Turner syndrome. Arch. Oral Biol..

[18] Funatsu M., Sato K., Mitani H. (2006). Effects of Growth Hormone on Craniofacial Growth: Duration of Replacement Therapy. Angle Orthod..

[19] Kjellberg H., Beiring M., Wikland K.A. (2000). Craniofacial morphology, dental occlusion, tooth eruption, and dental maturity. Oral Sci..

[20] Davidopoulou S., Chatzigianni A. (2017). Craniofacial morphology and dental maturity in children with reduced somatic growth of different aetiology and the effect of growth hormone treatment. Prog. Orthod..

[21] Wit J.M., Oostdijk W., Losekoot M., van Duyvenvoorde H.A., Ruivenkamp C.A.L., Kant S.G. (2016). Mechanism in endocrinology: Novel genetic causes of short stature. Eur. J. Endocrinol..

[22] MacGillivray M.H. (1987). Disorders of Growth and Development. Endocrinology and Metabolism.

[23] Poole A., Greene I., Buschang P. (1982). The effect of growth hormone therapy on longitudinal growth of the oral facial structures in children. Prog. Clin. Biol. Res..

[24] Takano K., Ogiuchi H., Hizuka N., Sangu Y., Shizume K. (1986). Oro-maxillofacial development in patients with GH deficiency and in normal short children. Endocrinol. Jpn..

[25] Cantu G., Buschang P.H., Gonzalez J.L. (1997). Differential growth and maturation in idiopathic growth-hormone-deficient children. Eur. J. Orthod..

[26] Bevis R.R., Hayles A.B., Isaacson R.J., Sather A.H. (1977). Facial growth response to human growth hormone in hypopituitary dwarfs. Angle Orthod..

[27] Isaksson O.G., Lindahl A., Nilsson A., Isgaard J. (1987). Mechanism of the stimulatory effect of growth hormone on longitudinal bone growth. Endocr. Rev..

[28] Rongen-Westerlaken C., Born E.V., Prahl-Andersen B., Teunenbroek A.V., Manesse P., Otten B., Tweel I.V., Kuijpers-Jagtman A., Delemarre vd Waal H., Drayer N. (1993). Effect of growth hormone treatment on craniofacial growth in Turner’s syndrome. Acta Paediatr..

[29] Forsberg C.-M. (2002). The effect of growth hormone therapy on mandibular and cranial base development in children treated with total body irradiation. Eur. J. Orthod..

[30] Gravholt C.H., Frystyk J., Flyvbjerg A., Ørskov H., Christiansen J.S. (2001). Reduced free IGF-I and increased IGFBP-3 proteolysis in Turner syndrome: Modulation by female sex steroids. Am. J. Physiol. Endocrinol. Metab..

[31] Gravholt C.H., Chen J.-W., Oxvig C., Overgaard M.T., Christiansen J.S., Frystyk J., Flyvbjerg A. (2006). The GH–IGF–IGFBP axis is changed in Turner syndrome: Partial normalization by HRT. Growth Horm. Igf Res..

[32] Rao E., Weiss B., Fukami M., Rump A., Niesler B., Mertz A., Muroya K., Binder G., Kirsch S., Winkelmann M. (1997). Pseudoautosomal deletions encompassing a novel homeobox gene cause growth failure in idiopathic short stature and Turner syndrome. Nat. Genet..

[33] Rao E. (2001). The Leri-Weill and Turner syndrome homeobox gene SHOX encodes a cell-type specific transcriptional activator. Hum. Mol. Genet..

[34] Marchini A., Marttila T., Winter A., Caldeira S., Malanchi I., Blaschke R.J., Häcker B., Rao E., Karperien M., Wit J.M. (2004). The Short Stature Homeodomain Protein SHOX Induces Cellular Growth Arrest and Apoptosis and Is Expressed in Human Growth Plate Chondrocytes. J. Biol. Chem..

[35] Blaschke R.J., Rappold G. (2006). The pseudoautosomal regions, SHOX and disease. Curr. Opin. Genet. Dev..

[36] Binder G., Baur F., Schweizer R., Ranke M.B. (2006). The d3-Growth Hormone (GH) Receptor Polymorphism Is Associated with Increased Responsiveness to GH in Turner Syndrome and Short Small-for-Gestational-Age Children. J. Clin. Endocrinol. Metab..

[37] Filipsson R., Lindsten J., Almqvist S. (1965). Time of eruption of the permanent teeth, cephalometric and tooth measurement and sulphation factor activity in 45 patients with Turner’s syndrome with different tyoes of X chromosome aberrations. Acta Endocrinol..

[38] Babic M., Glisic B., Scepan I. (1997). Mandibular growth pattern in Turner’s syndrome. Eur. J. Orthod..

[39] Dumancic J., Kaic Z., Varga M.L., Lauc T., Dumic M., Milosevic S.A., Brkic H. (2010). Characteristics of the craniofacial complex in Turner syndrome. Arch. Oral Biol..

[40] Babić M., Šćepan I., Mićić M. (1993). Comparative cephalometric analysis in patients with X-chromosome aneuploidy. Arch. Oral Biol..

[41] Grön M., Pietilä K., Alvesalo L. (1999). The craniofacial complex in 45, X/46, XX females. Arch. Oral Biol..

[42] Peltomäki T., Alvesalo L., Isotupa K. (1989). Shape of the craniofacial complex in 45, X females: Cephalometric study. J. Craniofac. Genet. Dev. Biol..

[43] Perkiomaki M.R. (2005). The relationship of distinct craniofacial features between Turner syndrome females and their parents. Eur. J. Orthod..

[44] Simmons K.E. (1999). Growth Hormone and Craniofacial Changes: Preliminary Data From Studies in Turner’s Syndrome. Pediatrics.

[45] Juloski J., Glisic B., Scepan I., Milasin J., Mitrovic K., Babic M. (2013). Ontogenetic changes of craniofacial complex in Turner syndrome patients treated with growth hormone. Clin. Oral Investig..

[46] Russell K.A. (2001). Orthodontic treatment for patients with Turner syndrome. Am. J. Orthod. Dentofac. Orthop..

[47] Cazzolla A.P., Lo Muzio L., Di Fede O., Lacarbonara V., Colaprico A., Testa N.F., Giuseppe T., Zhurakivska K., Marzo G., Lacaita M.G. (2018). Orthopedic-orthodontic treatment of the patient with Turner’s syndrome: Review of the literature and case report. Spec. Care Dent..

[48] Kang E.H., Yamaguchi T., Tajima A., Nakajima T., Tomoyasu Y., Watanabe M., Yamaguchi M., Park S.B., Maki K., Inoue I. (2009). Association of the growth hormone receptor gene polymorphisms with mandibular height in a Korean population. Arch. Oral Biol..

[49] Zhou J., Lu Y., Gao X., Chen Y., Lu J., Bai Y., Shen Y., Wang B. (2005). The growth hormone receptor gene is associated with mandibular height in a Chinese population. J. Dent. Res..

[50] Yamaguchi T., Maki K., Shibasaki Y. (2001). Growth hormone receptor gene variant and mandibular height in the normal Japanese population. Am. J. Orthod. Dentofac. Orthop..

[51] Dos Santos C., Essioux L., Teinturier C., Tauber M., Goffin V., Bougnères P. (2004). A common polymorphism of the growth hormone receptor is associated with increased responsiveness to growth hormone. Nat. Genet..

[52] Jorge A.A.L., Marchisotti F.G., Montenegro L.R., Carvalho L.R., Mendonca B.B., Arnhold I.J.P. (2006). Growth Hormone (GH) Pharmacogenetics: Influence of GH Receptor Exon 3 Retention or Deletion on First-Year Growth Response and Final Height in Patients with Severe GH Deficiency. J. Clin. Endocrinol. Metab..

